# Genome collinearity analysis illuminates the evolution of donkey chromosome 1 and horse chromosome 5 in perissodactyls: A comparative study

**DOI:** 10.1186/s12864-021-07984-6

**Published:** 2021-09-15

**Authors:** Shaohua Li, Gaoping Zhao, Hongmei Han, Yunxia Li, Jun Li, Jinfeng Wang, Guifang Cao, Xihe Li

**Affiliations:** 1 Research Center for Animal Genetic Resources of Mongolia Plateau, College of Life Sciences, Inner Mongolia University, Hohhot, 010070 China; 2 College of Basic Medicine, Inner Mongolia Medical University, Hohhot, 010110 China; 3 Inner Mongolia Saikexing Institute of Breeding and Reproductive Biotechnology in Domestic Animal, Hohhot, 011517 China; 4 Department of Physical Education, Hohhot Minzu College, Hohhot, 010051 China; 5 College of Veterinary Science, Inner Mongolia Agricultural University, Hohhot, 010018 China

**Keywords:** Chromosome-length genome assemblies, Comparative genomes, Chromosome evolution, DNA zoo

## Abstract

**Background:**

It is important to resolve the evolutionary history of species genomes as it has affected both genome organization and chromosomal architecture. The rapid innovation in sequencing technologies and the improvement in assembly algorithms have enabled the creation of highly contiguous genomes. DNA Zoo, a global organization dedicated to animal conservation, offers more than 150 chromosome-length genome assemblies. This database has great potential in the comparative genomics field.

**Results:**

Using the donkey (*Equus asinus asinus*, EAS) genome provided by DNA Zoo as an example, the scaffold N50 length and Benchmarking Universal Single-Copy Ortholog score reached 95.5 Mb and 91.6%, respectively. We identified the cytogenetic nomenclature, corrected the direction of the chromosome-length sequence of the donkey genome, analyzed the genome-wide chromosomal rearrangements between the donkey and horse, and illustrated the evolution of the donkey chromosome 1 and horse chromosome 5 in perissodactyls.

**Conclusions:**

The donkey genome provided by DNA Zoo has relatively good continuity and integrity. Sequence-based comparative genomic analyses are useful for chromosome evolution research. Several previously published chromosome painting results can be used to identify the cytogenetic nomenclature and correct the direction of the chromosome-length sequence of new assemblies. Compared with the horse genome, the donkey chromosomes 1, 4, 20, and X have several obvious inversions, consistent with the results of previous studies. A 4.8 Mb inverted structure was first discovered in the donkey chromosome 25 and plains zebra chromosome 11. We speculate that the inverted structure and the tandem fusion of horse chromosome 31 and 4 are common features of non-caballine equids, which supports the correctness of the existing *Equus* phylogeny to an extent.

**Supplementary Information:**

The online version contains supplementary material available at 10.1186/s12864-021-07984-6.

## Background

Resolving the evolutionary history of species genomes is important for our understanding of genome organization and chromosomal architecture [1]. Karyotype or chromosomal evolution has traditionally been inferred using classical and molecular cytogenetic approaches, such as comparisons of G-banded chromosome patterns and fluorescence in situ hybridization (FISH), with limited resolution [2–8]. FISH-based methods do not have sufficient resolution to permit accurate identification of evolutionary breakpoint regions and fine-scale rearrangements [7, 8]. Owing to the lowering of the cost associated with sequencing, an increasing number of species genome scaffolds have reached chromosome length, although contig N50 still needs to be improved. This allows the inference of most of the inversions, translocations, fusions, and fissions that occurred during evolution by simple observational comparisons on a sequence-based whole-genome scale [9, 10]. Nevertheless, we should realize that the genome assembly is not completely correct [11, 12]. Only relatively conservative structural variations or analytical results supported by experimental evidence at the molecular or cellular level are credible [12]. In addition, comparative genomic analysis at the sequence level is beneficial in revealing the details of variation [12]. Therefore, when we demonstrate the use of genome assembly data for exploring the chromosomal evolution, the selected chromosomes have an extensive basis of molecular cytology research, and for highlighting detailed advantages, the selected structural variations are more complex or small. The perissodactyl karyotypes are slowly evolving in ceratomorphs and extremely rapidly evolving in equids [6]. The comparative genomic research of this group is relatively thorough, especially in equine animals; region-specific painting and bacterial artificial chromosome probes are used to determine the direction of evolutionarily conserved segments with respect to centromere positions [6, 13, 14]. At present, the National Center for Biotechnology Information (NCBI) database only includes the chromosome-level genome assembly of horse and donkey in perissodactyls, whereas DNA Zoo has chromosome-level genomes of plains zebra, white rhinoceros and Malayan tapir. Therefore, we will focus on some chromosomes of perissodactyls, especially the horse and the donkey.

The domestic donkey, *Equus asinus asinus* (EAS, 2n = 62, NCBI Taxonomy ID: 83772), belongs to the Perissodactyla order, Equidae family, and *Equus* genus and descends from the African wild ass [15–17]. The order Perissodactyla includes three extant families: Tapiridae, Rhinocerotidae, and Equidae. The extant equid family is comprised of a single genus, *Equus*, which includes zebras, African and Asian asses, as well as horses. African and Asian asses are sister groups, and asses and zebras are also known as non-caballine equids [18]. The early interest in donkeys was primarily triggered by their cross-breeding with horses (*Equus caballus*, ECA), mules or hinnies, with heterosis and fertility in a few cases [19–25]. However, the exploration of the above-listed issues must be based on a deep understanding of the parents. Unlike the large number of studies available for horses, our understanding of donkeys is still relatively poor. Although the NCBI database currently contains chromosome-length donkey genome, the synteny relationship in several places is inconsistent with the results of previous FISH-based methods [6, 13, 14, 26]. For example, it is mentioned in its supplementary data 1 that the donkey chromosome 2 assembly has a homologous relationship with the horse chromosome 1 and 28, which is obviously inconsistent with the results of previous studies on the independent correspondence between donkey chromosome 2 and horse chromosome 1 [6, 13, 14, 26]. As a result, we decided to use the donkey assembly provided by DNA Zoo. 

DNA Zoo (https://www.dnazoo.org/), which is a global organization, dedicated to animal conservation, offers more than 150 chromosome-length genome assemblies of plants and animals [27, 28]. Here, we selected the donkey genome to prove the continuity and integrity of the assembly provided by this website. In addition, we identified the cytogenetic nomenclature, corrected the direction, and analyzed the genome-wide chromosomal rearrangements with reference to the results of the collinearity analysis performed in this study and previously published results. Finally, the donkey chromosome 1 and horse chromosome 5 were used as examples to explore their evolution in perissodactyls.

## Results

### Alignment to the horse genome and chromosome orientation

**Fig. 1:**
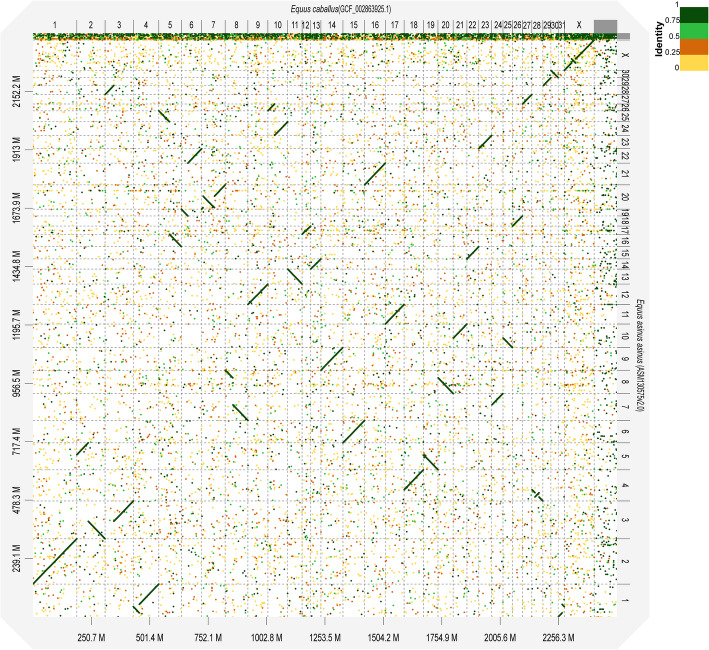
Dot-plot alignments of chromosomes of *E. caballus* (ECA) to *E. asinus asinus* (EAS) using D-Genies. The aligned segments are represented as dots or lines. The significant inversions included EAS1, EAS4, EAS20, and EASX, aligned to ECA 31 + 4, ECA 28, ECA 7, and ECA X, with sizes of 35, 17, 40, and 15 Mb, respectively. The colors correspond to similarity values that were binned in four groups (< 25, 25–50%, 50–75%, and > 75% similarity)

The present study identified an overall strong collinearity between the donkey and horse genomes (Figs. 1 and S1). Because the horse genome was constructed according to genetic physical mapping [11], we identified the cytogenetic nomenclature and adjusted the direction of the donkey chromosome-length sequence according to the subchromosomal painting results between donkey and horse [13, 29, 30]. Our findings showed the existence of 31 chromosome sequences numbered according to a cytogenetic standard, while 13 chromosome-length sequence directions were adjusted (including one scaffold internal adjustment). Finally, the modified donkey genome (ASM130575v2) was obtained and exhibited a scaffold N50 length of 95.5 Mb (Table 1). The comparison genome point diagram between the donkey and horse genomes is presented in Fig. 1. Compared with the horse genome, we observed a significant number of inverted structures in chromosomes 1, 4, 20, and X of the donkey, with sizes of 35, 17, 40, and 15 Mb, respectively. The inverted structure located inside the X chromosome was the first validation at the sequence level.

**Table 1:** Quality metrics for the present assembly when compared with previous donkey genome assemblies

	This study (improved DNA Zoo, ASM130575v2)	Huang et al. [31] (ASM130575v1)	Renaud et al. [12]
N50 contigs	66.7 kb	66.7 kb	140.3 kb
N50 scaffolds	95.5 Mb	3.8 Mb	15.4 Mb
Total bases	2.391Gb	2.391 Gb	2.320 Gb
Largest scaffold	185.41 Mb	17.06 Mb	84.20 Mb
Unresolved bases per 100 kb	1396.07	1384.93	1121.61
Total number of predicted protein-coding genes	18,937	23,850	18,984

### Gene annotation

**Fig. 2:**
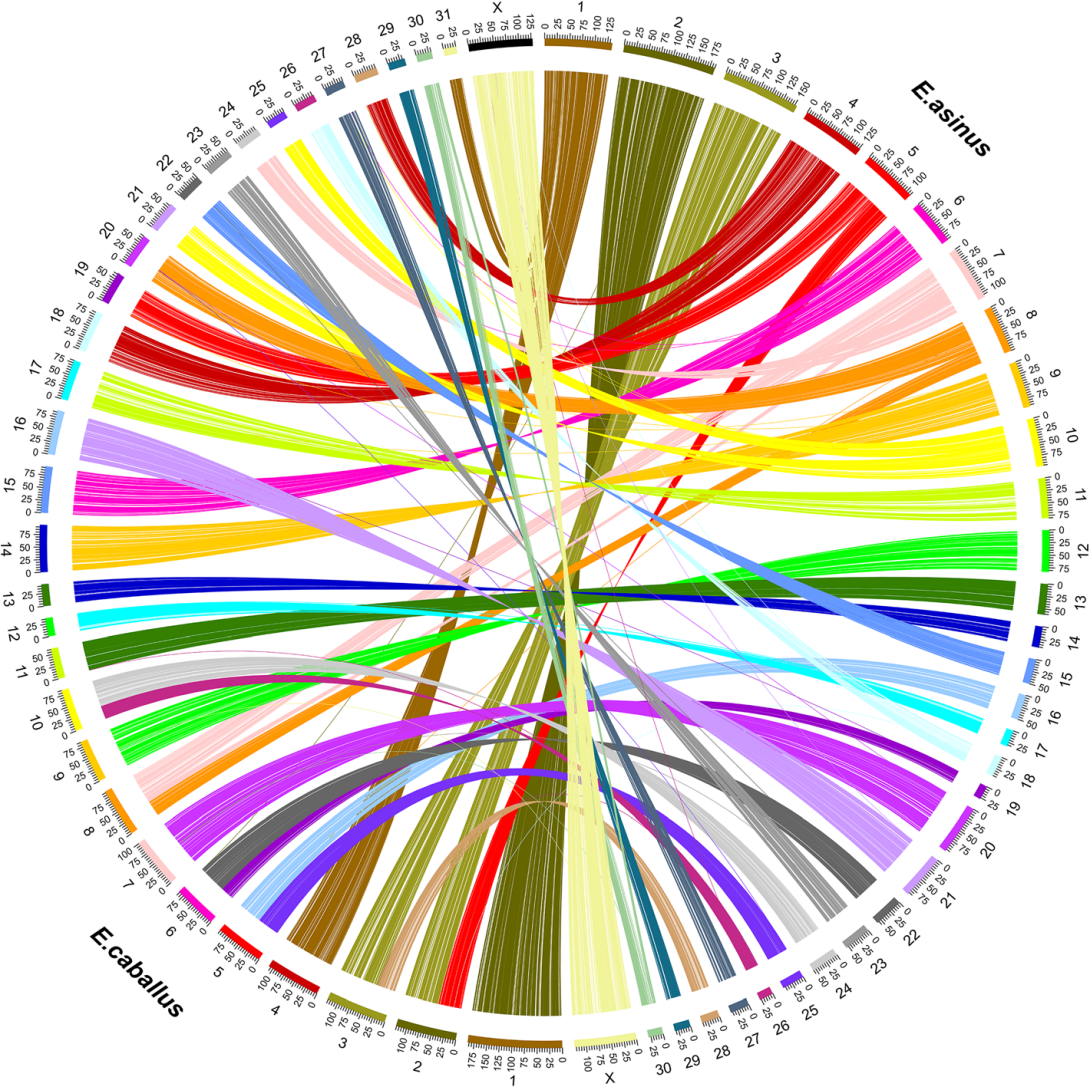
Circos plot of the collinearity analysis based on protein-coding genes between *E. asinus asinus* and *E. caballus*. The conservation of the gene order of all *E. asinus asinus* chromosomes to *E. caballus* matched the results of subchromosomal comparative mapping

**Fig. 3:**
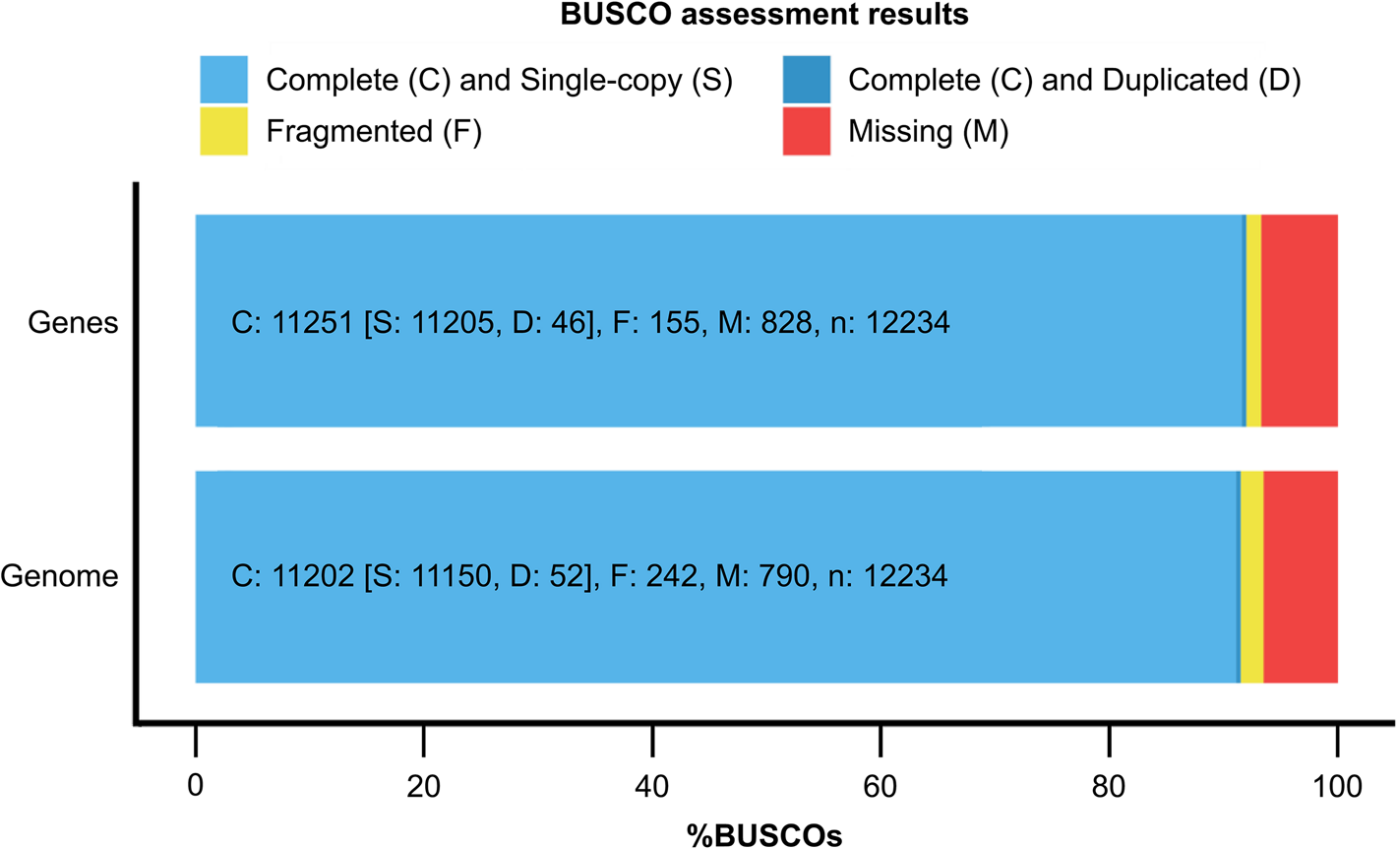
BUSCO completeness assessments for donkey genomics data quality control. The bar charts produced using the BUSCO plotting tool show proportions that were classified as complete (C, blue), complete single-copy (S, light blue), complete duplicated (D, dark blue), fragmented (F, yellow), and missing (M, red)

We predicted a total of 18,937 protein-coding genes through homology-based approaches and then used the annotations available for the horse to identify 16,691 donkey orthologs. A protein-based collinearity analysis showed the highly conserved order of genes for all chromosomes, as shown in Fig. 2. A Benchmarking Universal Single-Copy Ortholog (BUSCO) gene integrity analysis showed that among the lineage-specific profile (laurasiatheria_odb10.2019-11-20) which contains 12,234 markers, there were 11,205 single-copy orthologs and 46 duplicated orthologs, with complete genes accounting for 91.6% of these entities, which was indicative of good genomic integrity at the gene level (Fig. 3).

### Collinearity analysis of EAS1 and ECA5 in perissodactyls and outgroups

**Fig. 4:**
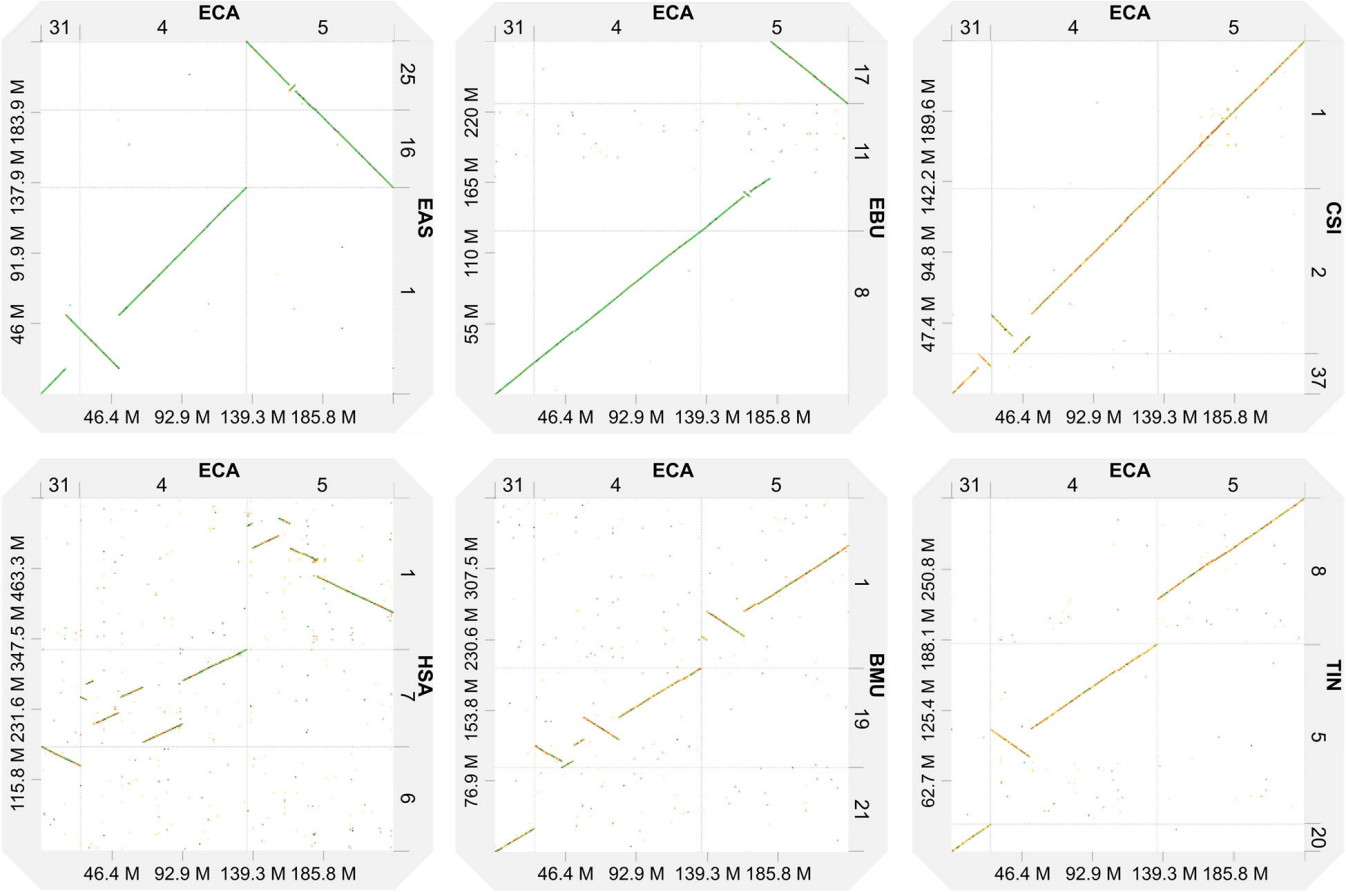
Dot-plot alignments of EAS1 and ECA5 and their orthologs. Animals involved in comparative genomics include zebra (*Equus burchellii*, EBU), white rhinoceros (*Ceratotherium simum*, CSI), Malayan tapir (*Tapirus indicus*, TIN), blue whale (*Balaenoptera musculus*, BMU), and humans (*Homo sapiens*, HSA). Because the orientations of the CSI, TIN, and BMU chromosome-length scaffolds are unknown a priori, they were oriented using the strand that minimized the number and size of inversions with respect to the horse chromosomes

**Fig. 5:**
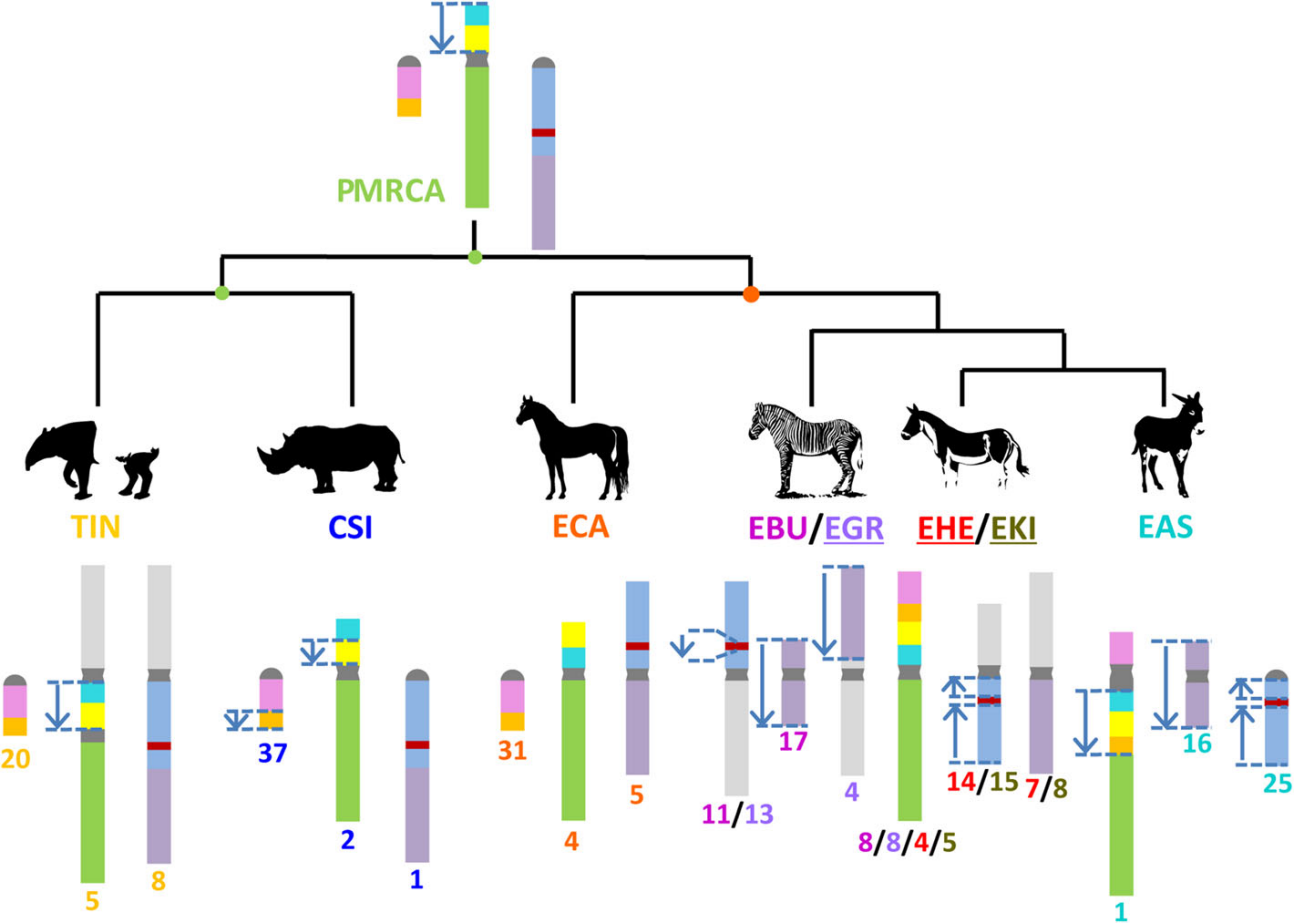
Inferred evolution diagram of the donkey chromosome 1 and horse chromosome 5 in perissodactyls. We built a cladogram based on previous research to help understand the evolution of EAS1 and ECA5. The results of subchromosomal comparative mapping and genomic collinearity analysis were summarized in this study using D-Genies and Mauve v2.4.0 among CSI, ECA, *Equus burchellii* (EBU), *Equus grevyi* (EGR), *Equus hemionus* (EHE), *Equus kiang* (EKI), and *Equus asinus asinus* (EAS). PMRCA represents the most recent common ancestor (MRCA) of odd-hoofed animals. Each colored region is a locally collinear block (LCB). The blue arrows indicate LCBs that were inverted when compared with horse orthologous regions. The number below the chromosome represents the cytogenetic nomenclature in the karyotype of different species. The underlined abbreviated species names indicate that the structural variation within the chromosome of the species is only inferred and needs to be supported by sequencing data. The light gray chromosome segments represent the fusion of the corresponding chromosomes and not homology

The horse genome has been used as a reference for describing the structural variation in all other species. The results of collinearity analysis showed that the association between the orthologous regions of ECA31 and ECA4 found in EAS and plains zebra (*Equus burchellii*, EBU) did not exist in the white rhinoceros (*Ceratotherium simum*, CSI), Malayan tapir (*Tapirus indicus*, TIN), blue whale (*Balaenoptera musculus*, BMU), and humans (*Homo sapiens*, HSA) (Figs. 4 and 5). An inversion and a shift were noted in EAS1, orthologous to ECA31 (Figs. 4 and 5). Chromosome translocation refers to the transfer of a portion of one chromosome to another position on the same chromosome or a nonhomologous chromosome. When a chromosome translocation occurs involving a single chromosome, it is referred to as a shift. The similar inversion breakpoint in EAS1, orthologous to ECA31, did not exist in the orthologous regions of EBU8, TIN20, BMU21, and HAS6 but was present in CSI37 (Figs. 4 and 5) [6, 13, 32]. We also detected an inversion and a shift in EAS1, CSI2, BMU19, and HAS7, orthologous to ECA4 (Figs. 4 and 5). The inversion breakpoint in EAS1, orthologous to ECA4, also occurred in BMU19, TIN5, and CSI2; however, because of the limited resolution of dot plots, it was not possible to determine the consistency of the inversion breakpoints or their evolution relationship. A collinearity analysis showed that the association between the short and long arms of ECA5, with the exception of EAS and EBU, was relatively conserved in perissodactyls; moreover, this conservation was also observed in humans among the order Primates and blue whales among the order Artiodactyla (Figs. 4 and 5). A 4.8 Mb inverted structure was first discovered in EAS25 and EBU11 (Figs. 1, 4 and 5).

We further used Mauve v2.4.0 for a more detailed analysis in ECA31 and 4 orthologous regions of perissodactyls. The results showed that the inverted breakpoints of EAS 1 and CSI37, orthologous to ECA31, were nearly located at the same position (Figures S2 and 5). Furthermore, the structure of the ECA4 orthologous regions was different among the various perissodactyl species. In the Malayan tapir, the ECA4p orthologous region is inverted; in the white rhinoceros, the region is inverted and shifted.

## Discussion

### DNA zoo remains unexplored

Using the HiC technology [27, 28], DNA Zoo based on NCBI draft data generated a new genome assembly for the domestic donkey [31]. Its scaffold N50 length reached 95.5 Mb, six times larger than the best previously published donkey assembly [12]. There were 18,937 annotated protein-encoding genes, and the BUSCO gene integrity evaluation reached 91.6%, which was close to Renaud’s genome assembly (18984) [12]. DNA Zoo offers chromosome-length genome assemblies of over 150 plants and animals. Considering the donkey genome alone as an example, the continuity and integrity of the assembly are relatively good and there is a good scope for exploration.

### Alignment to the horse genome

We found an overall strong collinearity between the donkey and horse genomes, a result that was consistent with those of a previous comparative genomic analysis performed using FISH [13, 14]. When compared with the ECA genome, we observed several distinct structural variations in chromosomes 1, 4, 20, and X of the donkey. Among them, EAS1, 20, and X were consistent with the results of a previous karyotype analysis and FISH [13, 33], whereas 1, 4, and 20 were consistent with the previous results of a subchromosomal genome assembly analysis [12]. The inverted structure detected within the X chromosome was the first validation at the sequence level. When compared with ECA5, a relatively small inverted structure (4.8 Mb) was found in EAS25 and EBU11. Such a small structural variation is difficult to screen using conventional molecular cytogenetic methods, such as karyotype analysis and FISH. Genetic or physical maps are relatively expensive and difficult to obtain. Therefore, based on the corresponding relationship between chromosome painting and gene mapping, we can refer to the genome assembly corrected by physical maps, such as the horse genome assembly, and orient chromosome-length sequences, eventually obtaining a chromosome-oriented assembly.

### Evolution of EAS1 in perissodactyls

Together with the previously published results of comparative chromosome maps in the perissodactyl species [6, 13], our results helped in reconstructing the evolution of EAS1 orthologs in perissodactyl main lineages, especially the evolution of the internal inverted structure (Fig. 5). A collinearity analysis showed that EAS1 was orthologous to ECA31 and ECA4, which was consistent with the results of previous research at the molecular cytological level. Moreover, this tandem fusion occurs only in non-caballine equids [6, 13]. The structure of ECA4 orthologous regions was different among the various perissodactyl species. Based on the phylogenetic relationship [34, 35] and parsimony principles, we speculated on the evolution of EAS1 orthologous regions in different perissodactyl lineages. In the perissodactyl most recent common ancestor (MRCA), the orthologous regions of ECA31 and ECA4 correspond to independent and complete chromosomes, respectively. Besides, this independence is consistent in all the existing perissodactyl species, except the non-caballine equids. When compared with the MRCA of the tapir family, in Malayan tapirs, the ECA4 orthologous region was fused with the ECA8q orthologous region. When compared with the MRCA of Rhinocerotidae, in white rhinoceros, the corresponding orthologous blocks to ECA31 and ECA4 were inverted once in the distal end of CSI37 and the short arm of CSI2, and the position of the inverted breakpoint of CSI37 was the same as the orthologous region of EAS1, which may imply the existence of structure variation hotspots in the relevant areas. When compared with the perissodactyl MRCA, in the *Equus* MRCA, the orthologous region of ECA4p was inverted, similar to that observed in the extant horse. In the non-caballine MRCA, ECA31 and ECA4 were fused in tandem, forming structures similar to those found in EBU8. Therefore, in the process of speciation of the African wild ass, the orthologous region of distal block of ECA31 and the ECA4p block as a whole were inverted (Figs. 4, 5 and S2). We would like to emphasize that, because of the poor resolution of the genome assembly in the centromeric regions, none of the abovementioned or following analyses involved relevant regions. All analyses were based on chromosome painting and genome assemblies of individuals, rather than on groups, and may lack generality. The results of collinearity analysis based on genome assembly may not be completely correct unless it is a conservative variation that appears multiple times; therefore, FISH experiments are needed to verify their credibility [12, 26].

### The direction of ECA5 evolution-fusion or fission

The long arm and the short arm of ECA5 are orthologous to chromosomes EPR23 (*Equus przewalskii*, EPR) and EPR24, respectively [6, 36, 37]. Myka and Ahrens et al. [36, 38] used karyotype analysis and FISH to establish that the karyotype difference between ECA and EPR was only a Robertsonian translocation. However, when compared with ECA5, whether the separation of EPR23 and EPR24 was an ancestral structure or a derived structure has not been determined. Trifonov and Piras et al. [6, 37] used chromosome painting and multiple rounds of probe analysis to reveal that the association between ECA5p and ECA5q is relatively conserved in rhinoceros and tapirs. However, their research could only prove the homology of the whole chromosome (arms) or the conservative relationship of the order of some detection points. The work could not address the collinearity of the whole chromosome and the subtle structural variation. A collinearity analysis based on a chromosome-length genome assembly showed that the association between the short arm and long arm of ECA5, except for Asian and African asses, was relatively conserved in perissodactyls (Figs. 4 and 5). Furthermore, this conservation was observed in humans among Primates and blue whales among Artiodactyla (Fig. 4). Based on the phylogenetic relationship and parsimony principles [34, 35], we inferred that the association between the short arm and long arm of ECA5 was an ancestral characteristic of perissodactyl species and that EPR23 and EPR24 were generated via a Robertsonian fission event accompanied by the speciation of *Equus przewalskii*. In addition, an inverted structure with a length of 4.8 Mb was detected in the orthologous region of ECA5 in EAS25 and EBU11; this may be a characteristic chromosomal structural variation that happened during the occurrence of non-caballine equids, which is similar to the abovementioned association of ECA31 and ECA4 (Figs. 4 and 5).

## Conclusions

Here we used the donkey genome as an example to prove the availability of DNA Zoo chromosome-level assembly data through the assessment of continuity and gene integrity and the collinearity analysis between horse and donkey genomes. By correcting the direction of chromosome-level sequence, we obtained a modified donkey assembly consistent with the previous results of FISH and sequence assembly.

When compared with the ECA genome, we observed several distinct inversions in donkey chromosomes 1, 4, 20, and X, which were also consistent with previous research results. Among them, to our knowledge, the inverted structure within the X chromosome was the first validation at the sequence level.

In addition, we reported the evolutionary details of EAS1 and ECA5 in perissodactyls using the chromosome-level genome assembly data provided by DNA Zoo and NCBI databases. ECA31 and 4 are the orthologous chromosome of EAS1. In the perissodactyl MRCA, ECA31 and 4 are independent. This independence also exists in Tapiridae and Rhinocerotidae. We speculate that ECA4p was inverted in the Equus MRCA, and then the tandem fusion of ECA31 and 4 occurred in non-caballine equids. The tandem fusion of ECA31 and 4 is a possible feature of MRCA of non-caballine equids at the chromosome level.

Moreover, the short arm of ECA5 is orthologous to EAS25, and the long arm is orthologous to EAS16. The association between the short and long arms of ECA5 is found in both Tapiridae and Rhinocerotidae. We speculate this association to be present in MRCA of perissodactyls and equines too. In non-caballine equids, the long and short arms of ECA5 are broken to form independent chromosomes. This independence was retained in African asses, while different forms of chromosomal fusion occurred in zebras and Asian asses.

We discovered a 4.8 Mb inverted structure for the first time within the EAS25 and EBU11 compared to ECA. We speculate that the inverted structure is a common feature of non-caballine equids, proving the correctness of the existing equine phylogeny from the chromosome level [34, 35]; this also shows that the detailed analysis of sequence-based karyotype evolution has certain significance in predicting phylogenetic relationships.

With the lowering of the sequencing costs, an increasing number of chromosome-level genomes will be published. Combined with chromosome painting and gene mapping, the comparative genome based on chromosome-length genome assembly is bound to yield more interesting findings in the study of chromosome evolution.

## Methods

### Sampling of species genome assemblies

In the NCBI Genome database, among equids, the horse genome assembly alone has currently reached chromosome level. We collected the chromosome-length genome assemblies of plains zebra, white rhinoceros (*Ceratotherium simum*, CSI), Malayan tapir (*Tapirus indicus*, TIN), blue whale (*Balaenoptera musculus*, BMU), and humans (*Homo sapiens*, HSA) from the NCBI and DNA Zoo databases (Table S1) [27].

### Chromosome orientation

The comparative genome point map between the horse and donkey genomes was generated using D-Genies (default parameters) [39]. Subsequently, BEDTools was used to modify the sequence direction of chromosomes according to the horse chromosome sequence direction and chromosome painting results [40].

### Genome quality metrics

Various quality metrics were computed to evaluate the modified genome continuity using QUAST V5.0.2 (default parameters), including N50 and other technical metrics [41]. Based on the evolutionarily informed expectations of the gene content of near-universal single-copy orthologs, the BUSCO v4.0.6 metric is complementary to technical metrics, such as N50. The laurasiatheria_odb10 lineage-specific profile, which contains 12,234 BUSCO markers, was tested against assemblies of the donkey using the option “-m geno” [42].

### Repeat masking for gene annotation

Before gene annotation, repeat masking was performed. RepeatMasker v4.0.9 was used to identify the transposable elements and tandem repeats that matched the entries in the Dfam_3.1 and RepBase-20,181,026 repeat library and were masked out in a soft-masked manner using the option “-parallel 20 -species equus -xsmall” [43].

### Gene annotation

The homologous annotation approach based on the comparative genome was used to annotate the modified donkey genome. Cactus v1.1.1 [44] was used to generate the comparison genome file, CESAR v2.0 [45] was used as the annotation software, and the NCBI database annotation file “GCF_001305755.1_ASM130575v1_genomic.gff.gz” was used as the reference annotation. The guide tree was “(ECA:0.0033,(ASM130575v1:0.00023, ASM130575v2:0.00023):0.00303).”

The resulting gene set was used to identify the corresponding homologs in the horse genome. Orthologs in the horse genome were obtained using OrthoFinder v2.4.0 [46] with default parameters. The parsing of the output obtained in OrthoFinder was performed in-house using custom scripts. The software instructions suggested the presence of sufficient species sampling for inferring a good phylogenetic tree. Accordingly, six species with relatively complete genome annotations were selected to identify and analyze of the orthologs. Exception for ECA and EAS, the other species were used as outgroups (cattle, pigs, mice, and humans), as detailed in Table S2.

Circos v0.69–8 was used to visualize the collinearity relationships of orthologs between ECA and EAS.

### Comparative genomic analysis

We used D-Genies (default parameters) to generate Dot-plot alignments between the horse and donkey genomes [39]. In addition, Mauve V2.4.0 (default parameters) was used for the visualization and detailed analysis of the study results [47].

## Supplementary Information

**Additional file 1.**

## Data Availability

The datasets generated during and/or analyzed during the current study are available from the website (DOI: 10.6084/m9.figshare.14387126).

## Abbreviations

BMU: *Balaenoptera musculus*
BUSCO: Benchmarking Universal Single-Copy Ortholog
CSI: *Ceratotherium simum*
EAS: *Equus asinus asinus*
EBU: *Equus burchellii*
ECA: *Equus caballus*
EGR: *Equus grevyi*
EHE: *Equus hemionus*
EKI: *Equus kiang*
EPR: *Equus przewalskii*
FISH: Fluorescence in situ hybridization
HAS: *Homo sapiens*
LCB: Locally collinear block
MRCA: Most recent common ancestor
TIN: *Tapirus indicus*
