# Supplementary material for: Genome collinearity analysis illuminates the evolution of donkey chromosome 1 and horse chromosome 5 in perissodactyls: A comparative study
Source: BMC Genomics. 2021 Sep 15;22:665. doi: 10.1186/s12864-021-07984-6 (PMC8442440; doi:10.1186/s12864-021-07984-6)
Supplement: Supplementary file 1 — Additional file 1. [file 12864_2021_7984_MOESM1_ESM.docx]

**Genome collinearity analysis illuminates the evolution of donkey chromosome 1 and horse chromosome 5 in perissodactyls: A comparative study**

Shaohua Li,^a,b,c^ Gaoping Zhao,^c^ Hongmei Han,^a,c^ Yunxia Li,^a,c^ Jun Li,^c^ Jinfeng Wang,^b^ Guifang Cao,^d^ Xihe Li^a,c*^

^a^ Research Center for Animal Genetic Resources of Mongolia Plateau, College of Life Sciences, Inner Mongolia University, Hohhot 010070, China

^b^ College of Basic Medicine, Inner Mongolia Medical University, Hohhot 010110, China

^c^ Inner Mongolia Saikexing Institute of Breeding and Reproductive Biotechnology in Domestic Animal, Hohhot 011517, China

^d^ College of Veterinary Science, Inner Mongolia Agricultural University, Hohhot 010018, China

***Corresponding author**

Xihe Li

Research Center for Animal Genetic Resources of Mongolia Plateau, College of Life Sciences, Inner Mongolia University, Hohhot 010070, China

Phone Number: 1-533-555-7993

Fax No.: Unavailable

E-mail address: [lixh@imu.edu.cn](mailto:lixh@imu.edu.cn)

**Supplementary Figure 1**

Dot-plot alignments of the 32 chromosomes of *E. caballus* (ECA, x-axis) to the 31chromosome-length scaffolds of *E. asinus asinus* (EAS, y-axis) using D-Genies (default parameters). The chromosome-length scaffolds of donkey were offered by DNA Zoo. The colors correspond to similarity values that were binned in four groups (<25%, 25%–50%, 50%–75%, and >75% similarity). This diagram depicts the direction of the genome sequence prior to adjustment.


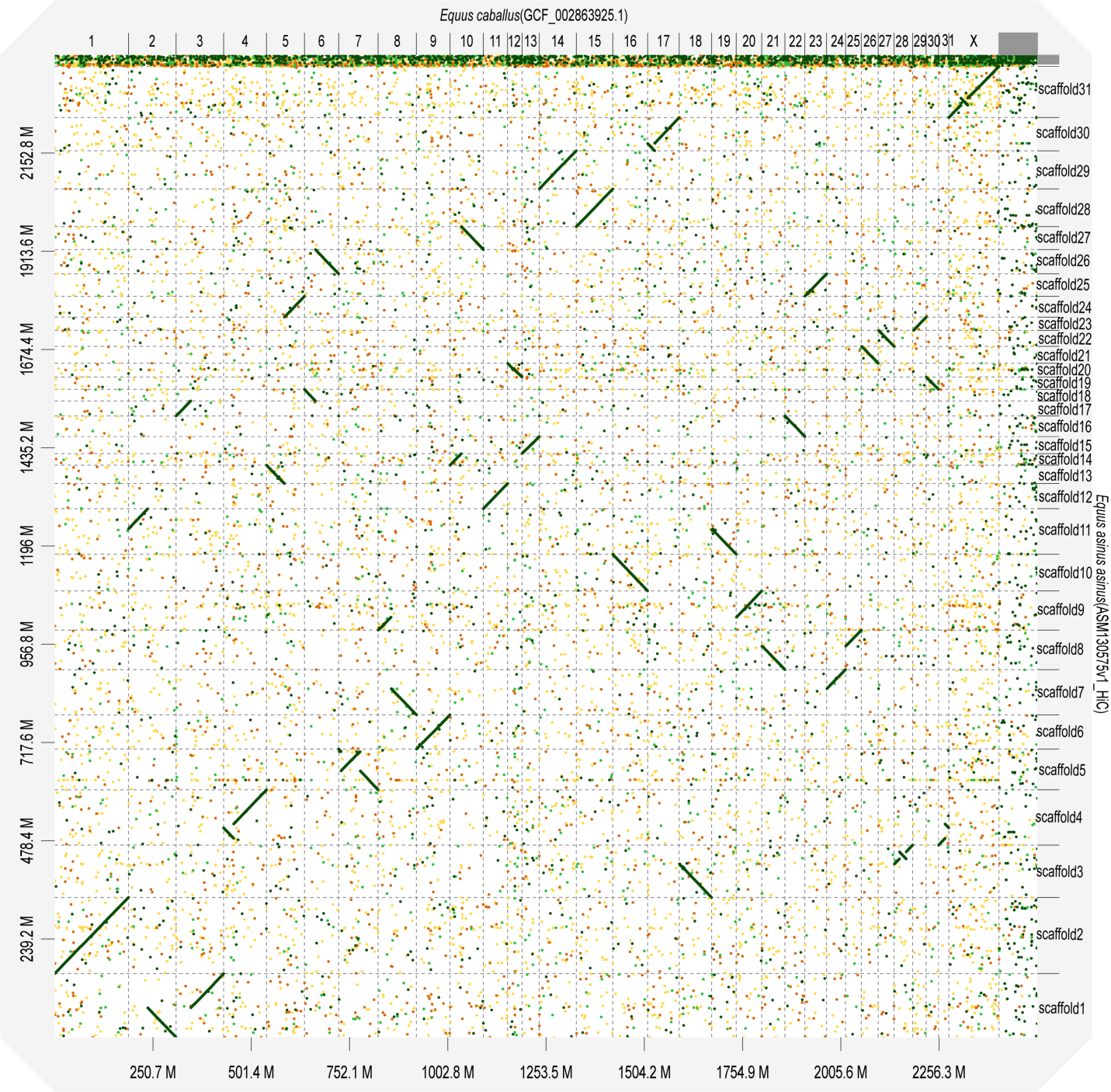


Supplementary Figure 2

Inverted breakpoint detailed analysis of EAS1 using Mauve v2.4.0. Each contiguously colored region is a locally collinear block (LCB), which is a region without rearrangement of the homologous backbone sequence. LCBs below a genome’s center line are in the reverse complement orientation relative to the reference genome. The lines between genomes trace each orthologous LCB through each of the genomes. The diagram shows detailed structural variations in the genome using Mauve V2.4.0; color boxes represent orthologous blocks.


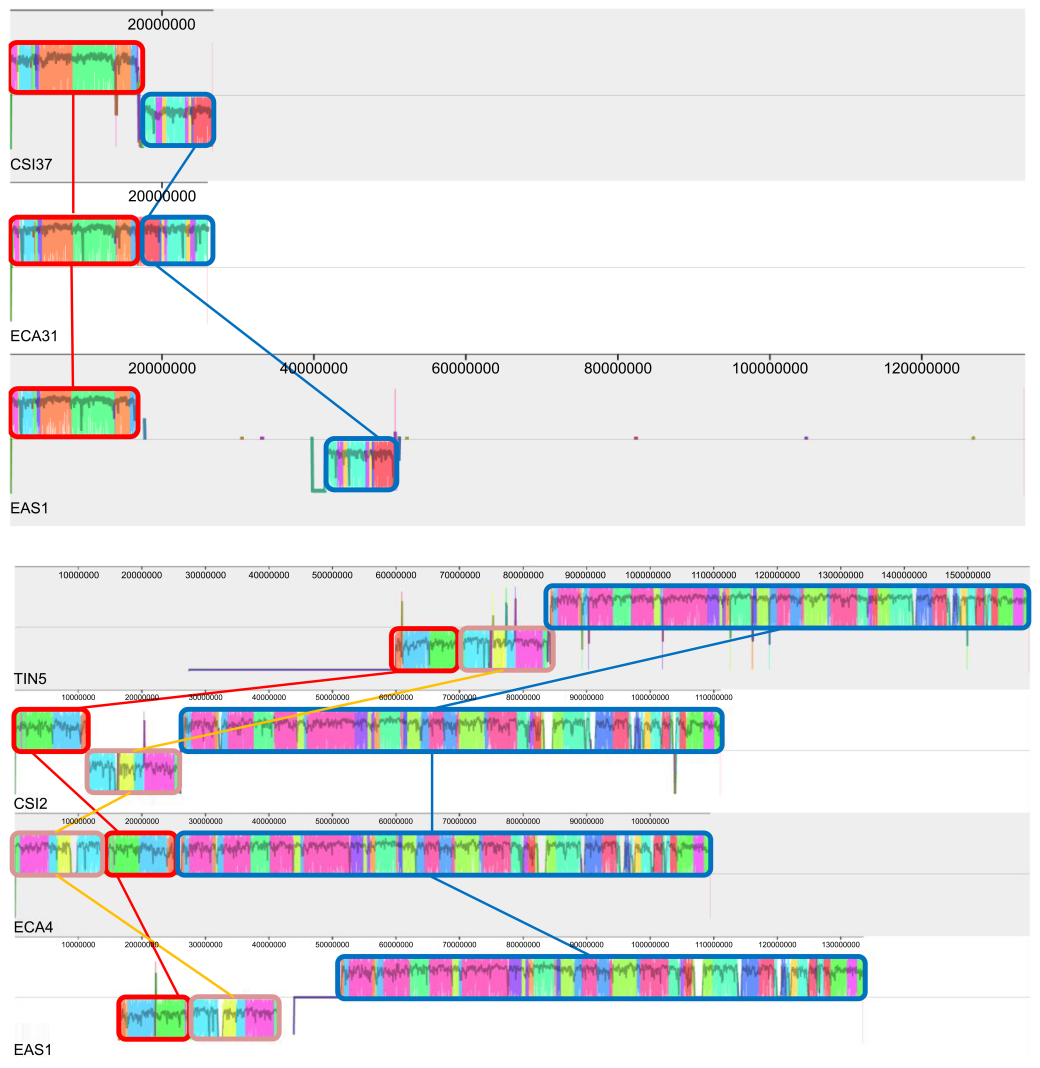


Supplementary Table 1: Genomic assembly data sources. Links to genome assemblies file website

| Species | Abbreviation | Common name | 2n | Database | Website link | |
| --- | --- | --- | --- | --- | --- | --- |
| *Equus asinus asinus* | EAS | Donkey | 62 | DNA ZOO | https://www.dropbox.com/s/my36jgqte50j2sc/ASM303372v1_HiC.fasta.gz?dl=0 |  |
| *Equus caballus* | ECA | Horse | 64 | NCBI | https://ftp.ncbi.nlm.nih.gov/genomes/all/GCF/002/863/925/GCF_002863925.1_EquCab3.0/GCF_002863925.1_EquCab3.0_genomic.fna.gz |  |
| *Equus burchellii* | EBU | Plains zebra | 44 | DNA ZOO | https://www.dropbox.com/s/tormwrmtzhayrc5/Equus_quagga_HiC.fasta.gz?dl=0 |  |
| *Ceratotherium simum* | CSI | white rhinoceros | 82 | DNA ZOO | https://www.dropbox.com/s/oqm312vygoh3csn/CerSimSim1.0_HiC.fasta.gz?dl=0 |  |
| *Tapirus indicus* | TIN | Malayan tapir | 52 | DNA ZOO | https://www.dropbox.com/s/vmq9iudk5cuyte0/Tapirus_indicus_HiC.fasta.gz?dl=0 |  |
| *Balaenoptera musculus* | BMU | Blue whale | 38 | NCBI | https://ftp.ncbi.nlm.nih.gov/genomes/all/GCA/009/873/245/GCA_009873245.2_mBalMus1.v2/GCA_009873245.2_mBalMus1.v2_genomic.fna.gz |  |
| *Homo sapiens* | HSA | Human | 46 | NCBI | https://ftp.ncbi.nlm.nih.gov/genomes/all/GCF/000/001/405/GCF_000001405.39_GRCh38.p13/GCF_000001405.39_GRCh38.p13_genomic.fna.gz |  |

Supplementary Table 2: Genome annotation data sources. Links to genome annotation file website

| Species | Abbreviation | Common name | Database | Website link |
| --- | --- | --- | --- | --- |
| *Equus asinus asinus* | EAS | Donkey | NCBI | ftp://ftp.ncbi.nlm.nih.gov/genomes/all/GCF/001/305/755/GCF_001305755.1_ASM130575v1/GCF_001305755.1_ASM130575v1_genomic.gff.gz |
| *Equus caballus* | ECA | Horse | NCBI | https://ftp.ncbi.nlm.nih.gov/genomes/all/GCF/002/863/925/GCF_002863925.1_EquCab3.0/GCF_002863925.1_EquCab3.0_genomic.gff.gz |
| *Bos taurus* | BTA | Cattle | NCBI | https://ftp.ncbi.nlm.nih.gov/genomes/all/GCF/002/263/795/GCF_002263795.1_ARS-UCD1.2/GCF_002263795.1_ARS-UCD1.2_genomic.gff.gz |
| *Sus scrofa* | SSC | Pig | NCBI | ftp://ftp.ncbi.nlm.nih.gov/genomes/all/GCF/000/003/025/GCF_000003025.6_Sscrofa11.1/GCF_000003025.6_Sscrofa11.1_genomic.gff.gz |
| *Mus musculus* | MMU | House mouse | NCBI | https://ftp.ncbi.nlm.nih.gov/genomes/all/GCF/000/001/635/GCF_000001635.26_GRCm38/GCF_000001635.26_GRCm38.p6_genomic.gff |
| *Homo sapiens* | HSA | Human | NCBI | https://ftp.ncbi.nlm.nih.gov/genomes/all/GCF/000/001/405/GCF_000001405.39_GRCh38.p13/GCF_000001405.39_GRCh38.p13_genomic.gff.gz |
